# AI in Medical Imaging and Image Processing

**DOI:** 10.3390/jcm14124153

**Published:** 2025-06-11

**Authors:** Karolina Nurzynska, Michał Strzelecki, Adam Piórkowski, Rafał Obuchowicz

**Affiliations:** 1 Algorithmics and Software Division, Silesian University of Technology, 44-100 Gliwice, Poland; 2 Institute of Electronics, Lodz University of Technology, 90-924 Lodz, Poland; michal.strzelecki@p.lodz.pl; 3 Department of Biocybernetics and Biomedical Engineering, AGH University of Krakow, 30-059 Krakow, Poland; pioro@agh.edu.pl; 4 Department of Diagnostic Imaging, Jagiellonian University Medical College, 31-501 Krakow, Poland; r.obuchowicz@gmail.com

Artificial intelligence methods have evolved sufficiently to be widely applied in automatic data analysis, providing standardized and reproducible results comparable to those of highly skilled specialists, while assisting less experienced personnel. This allows for a reduction in repetitive work and draws attention to the choice of medical treatment and accurate patient cases. Convolutional neural networks, attention-based transformers, and traditional machine learning approaches using radiomics features are at the forefront of medical applications, where the automatic understanding of various imaging techniques is needed (such as computed tomography—CT, magnetic resonance imaging—MRI, X-ray, radiographs, and positron emission tomography—PET) to examine internal organs and tissues. Moreover, graph neural networks are also making strides in this field. Finally, rapid progress in large language models has led to the integration of speech recognition and text analysis technologies, further streamlining radiologists’ workflows [1].

This collection of research highlights the influence of artificial intelligence (AI) and machine learning (ML) on healthcare, demonstrating their capacity to improve diagnostics, treatment strategies, and patient care. The presented studies address critical healthcare challenges, offering solutions that enhance precision, productivity, and healthcare accessibility. The compilation includes works on diagnostic improvement, featuring models such as convolutional neural networks (CNNs) and transformers for the early and accurate identification of various conditions, including different types of cancer, stroke, intracranial hemorrhage, and acute aortic syndrome (AAS). AI’s integration into radiology is showcased, facilitating tasks like the automatic segmentation of anatomical structures, cancer prediction using radiomics, and image quality optimization, thereby enhancing workflow efficiency and consistency. The collection also presents AI applications in surgical planning and intraoperative guidance, as evidenced by studies evaluating preoperative imaging predictors and AI tools for detecting surgical wound infections. These advancements enable personalized medicine by customizing interventions based on individual patient characteristics. Innovative technologies such as microwave imaging for breast cancer detection and steady-state thermal imaging are featured, illustrating AI’s role in developing non-invasive diagnostic methods that offer safer and more accessible alternatives to conventional techniques. The compilation also includes insights into AI-driven tools designed to track disease progression in chronic conditions and monitor postoperative recovery. Novel AI methodologies addressing rare and complex diagnoses, such as early-stage osteosarcoma detection, bone mineral density screening in cystic fibrosis, and biomarker identification in leukemia, are presented. The collection incorporates articles on AI-powered tools for emergency settings, capable of detecting rib fractures, coronary occlusion, or chest abnormalities, ensuring effective patient management in critical scenarios. Studies focusing on tuberculosis and COVID-19 detection from radiographs emphasize AI’s potential in global health applications, reflecting the development of AI-powered tools aimed at democratizing healthcare. The compilation also addresses practical aspects of AI integration, such as interrater variability, reproducibility, and the need for standardized benchmarks. This comprehensive collection serves as a valuable resource for healthcare professionals, researchers, and technologists seeking to understand and leverage AI’s potential in medicine. By combining in-depth technical insights with practical clinical applications, these studies contribute to improving patient outcomes, streamlining workflows, and promoting a more equitable healthcare system.

Deep learning models can be valuable when quick assessment of chest X-rays is needed to differentiate between acute aortic syndrome and thoracic aortic aneurysm without expert personnel available, as indicated in [2]. Employing a four-view network based on EfficientNet and incorporating an attention module could enhance breast cancer diagnosis from mammograms [3]. Implementing data augmentation techniques and developing novel network architectures may substantially improve the detection of tuberculosis and COVID-19 in CT scans [4]. Selecting the appropriate hyperparameters can optimize deep model performance, as shown in the case of brain tumor classification using MRI data [5].

Object localization within medical data can be accomplished through various methods. The most prevalent approach involves semantic segmentation using the U-Net network architecture, which assigns each pixel to a specific class. For example, when examining H&E (haematoxylin and eosin)-stained images, an accurate cell count across different classes is crucial for proper diagnosis, making precise segmentation, often achieved through U-Net architecture, essential [6]. The U-Net model facilitates the identification of heart and lung anatomy in chest X-rays, necessary for cardiothoracic ratio calculation, as shown in [7]. It can also be employed for the precise segmentation of the appendix with distinct boundaries, critical for diagnosing acute appendicitis from CT scans [8]. The determination of temporomandibular joint placement in ultrasonography images is explored in [9]. U-Net networks enhanced with pyramid attention modules and preprocessed with stick filter derivatives demonstrate 91–93% accuracy in lung fissure segmentation in CT images [10]. Despite its power, the U-Net architecture has limitations. In [11], researchers propose augmenting features derived from this model with a graph neural network to better capture the spatial relationships of features significant in COVID-19 diagnosis from CT scans. Moreover, repurposing existing datasets for different applications is challenging. In [12], researchers propose an algorithm to convert the original three-label annotation in the BraTS dataset to two-label annotations suitable for post-operative settings, which were then used to train the nnU-Net for brain segmentation in treatment response monitoring. The same network architecture proved to yield very good outcomes, with an F1-score of 94% for the automatic segmentation of the nasolacrimal canal in CBCT (cone beam computed tomography) imaging [13]. Ref. [14] introduces a novel semantic segmentation network for brain tumor annotation within the BraTS dataset, achieving 88–93% segmentation accuracy depending on the category. It is noteworthy that determining the training methodology can be complex, as the number of classes used may influence the outcomes, as demonstrated in research on osteosarcoma in X-rays and normal radiographs, where using more classes for model training yielded better quality results [15]. An alternative approach to object detection employs variations in Mask R-CNNs to determine approximate rectangular region boundaries. Enhancing mask detection with Mask R-CNN enables high-quality appendix segmentation in CT scans, as evidenced in [16]. Utilizing Faster R-CNN with the Feature Pyramid Network allows for rib fracture detection on chest radiographs with 89% accuracy [17].

Convolutional neural networks have numerous potential applications beyond those already mentioned. The detection of anomalies in optical coherence tomography (OCT) images using local region analysis has demonstrated 99% accuracy [18]. Denoising techniques are valuable for enhancing the quality of the medical data obtained from non-contrast chest and low-dose abdominal CT scans, as the image quality can vary depending on the equipment used [19]. Research presented in [20] indicates that employing denoising methods can particularly improve the output of older imaging devices. In the context of diffusion tensor imaging, while deep learning denoising techniques reduce femorotibial factorial anisotropy regardless of voxel size, these approaches may help to address the challenge of lower signal-to-noise ratios [21]. A broad review of various techniques concerning coronary artery calcium scoring on non-dedicated CT is described in [22]. An application of CNNs for pediatric neuroimaging presents the potential of this approach in [23].

Recent advancements have shown that attention mechanisms and transformer networks are not only effective in automated text analysis, but also yield impressive results in image processing. For example, [24] introduced the Vertebrae-aware Vision Transformer to ensure precise and efficient spine segmentation from CT scans. A transformer-based model was employed for bone age estimation from radiographs, achieving a mean absolute error of 4.1 months [25], which surpasses the traditional machine learning methods outlined in [26]. The tedious task of manually analyzing H&E-stained WSI (whole slide images) can now be replaced by an attention-based network, enhancing accuracy and subsequently reducing the number of required surgeries [27]. The Efficient Spatial Channel Attention Network for breast cancer detection in histopathological images has demonstrated superior performance compared to conventional convolutional neural networks, attaining 94% accuracy [28]. Transformers used in combination with the inverse Fourier transform enhance the performance of the convolution layer in rosette trajectory magnetic resonance imaging [29].

Numerous organizations opt to enhance their medical data acquisition equipment with artificial intelligence-based analysis software. The research outlined in [30] indicates that an AI-powered ECG (electrocardiogram) biomarker could identify coronary occlusion in resuscitated patients, performing comparably to an expert consensus. The application of AI systems for detecting dental caries enables the establishment of a gold standard and minimizes discrepancies among dentists’ assessments [31]. The findings presented in [32] demonstrate that implementing an AI-based system for identifying limb fractures in radiographs decreased the overall disagreement between physicians. An AI-driven system for screening low bone mineral density in low-dose chest CT scans proves valuable [33], as does AI support in OCTA (optical coherence tomography angiography) analysis for diagnosing various medical conditions [34]. These AI models can also streamline effective dose calculations in PET/CT, eliminating variations between operators [35]. There are also approaches on how to create simple, mobile applications that could enhance physician work. In [36], a RedScar mobile application for post-operative scar healing condition determination is introduced. The newly suggested, highly sensitive imaging technique proved to better detect breast cancer compared to MRI and infrared imaging [37]. The conclusions in [38] indicate that collaborative analysis and discussion accelerate the achievement of more accurate results when humans are learning to use new equipment.

The effectiveness of radiomic features in describing image content has been demonstrated, enabling further data classification. Research in [39] indicates that when extracted from CT or MRI scans of lung or prostate tumors, these features are influenced by individual variations in contrast. This underscores the importance of normalizing data before applying such methods [40]. Conversely, radiomic information derived from the MRI T2W modality yields more standardized results, potentially enhancing PIRADS (prostate imaging reporting data system) scores in a broader context [41]. Texture analysis, specifically gray-level co-occurrence matrix parameters, has shown a strong correlation with the histopathological grades of head and neck squamous cell carcinoma, emerging as an age-independent marker according to the study presented in [42]. A comparison of several traditional classification methods based on radiomic features with convolutional neural networks, trained both with and without transfer learning, was conducted to reduce the false-positive rates in prostate cancer detection using MRI images. The findings reveal that the tumor location affects the performance of all approaches, though deep learning models appear to be less susceptible to this issue [43].

A system for categorizing patients with idiopathic macular holes into those with favorable or unfavorable vision six months post vitrectomy can be developed using logistic regression, based on carefully chosen features from OCT data and medical records [44]. In another study, logistic regression and XGBoost were employed on features extracted via casual forest from functional MRI time series data, achieving 97% accuracy in distinguishing between healthy individuals and Parkinson’s disease patients [45]. A comparable approach demonstrated the effectiveness of the SAFE (scan and find early) microwave imaging device in detecting breast cancer in young women (83%) and those with dense breasts (91%) [46]. Various machine learning models were assessed to identify an accurate model for dose estimation in CT [47].

Statistical analyses reveal that the left atrial volume and function derived from four-chamber measurements are equivalent to biplane methods when appropriate bias correction factors are applied [48]. Preoperative CT findings correlate with the duration of laparoscopic appendectomy in pediatric cases, as demonstrated in [49]. Research has also shown that categorizing osteophytes into four distinct types based on CT scans impacts the planning of surgical interventions [50]. Statistical analysis of multi-color flow cytometry data using t-SNE (t-distributed stochastic neighbor embedding) enabled the identification of multiple clusters in leukemic cells, based on their antigen expression composition and intensity [51]. K-means analysis was applied to determine the separation of the total kidney volume in patients treated with tolvaptan and allowed it to be shown between others that high annual growth was associated with a responder phenotype [52].

Utilizing fully labeled data to train models enables the resolution of numerous challenges in automated medical data analysis. However, the process of creating annotations is time-consuming and costly, necessitating the exploration of alternative methods when labels are unavailable. In such cases, unsupervised algorithms prove valuable. For instance, research in [53] demonstrates that employing non-linear dimensionality reduction techniques, such as locally linear embedding and isometric feature maps, facilitates the identification of active lesions in MRI scans during the planning of multiple sclerosis diagnosis and treatment. A study in [54] introduces a k-means approach for detecting and quantifying immunohistochemical staining. This method, when applied to the tissue of interest with normalized color and a removed extraneous background, yields good outcomes.

Transformer networks emerged as a solution to various challenges in automated text processing and comprehension. When trained on extensive datasets, these models demonstrate good capabilities in swift knowledge acquisition and, through generative approaches, can operate across different modalities. Consequently, their application as a supportive tool in medicine is a logical choice. For example, in medical imaging analysis for diagnosis and treatment planning, findings must be documented textually, forming the basis of communication. To streamline this process, an automatic speech recognition system can be employed, as shown in [55]. Advanced language models such as GPT-4 can automatically describe the imaging data content, serving as an initial step towards assisting radiologists in image description. However, using these models without proper preparation leads to high error rates, while employing the current prompts can enhance results, as outlined in [56], along with appropriate tuning [57]. In [58], researchers compare GPT-4o with Cloud 3.5 Sonnet models for the automatic detection of acute ischemic stroke in diffusion-weighted imaging, revealing superior diagnostic abilities in the latter model, albeit still noting a high error rate that necessitates further investigation.

Table 1 summarizes the application of various AI approaches to medical data. Within the publication cycle, three main areas related to the topic of artificial intelligence in medicine can be distinguished. The largest group of articles (24) is the implementation of systems in specific medical issues, mainly using deep learning, U-Net, or CNNs. Most of them are studies of the proposed architecture for one problem, but there are also four publications testing and comparing different architectures. Three of the publications concern histopathological images. A very frequently discussed issue in this group of publications is image segmentation applied to a wide class of organs and tissues. A summary of such papers is presented in Table 2.

The second largest group of publications (12) concerned the use of artificial intelligence systems for the classification of medical data. The most commonly used method was logistic regression, but other regressors were also used, e.g., random forest, and once the stochastic t-SNE method was used. A summary of topic papers focused on pathology detection and classification is shown in Table 3.

The third group of publications are analyses of the use of existing software based on artificial intelligence (e.g., The Quantitative ECG (QCG™) system, PixelShine, or A.I. DeNoise™). The cycle also contains three review papers.

Figure 1 illustrates the distribution of imaging modalities used in publications within this topic. CT and MRI are predominant, which is expected given their widespread use across medical specialties. This correlates with the number of commercially available AI solutions for CT and MRI [1], representing the modalities with the most developed image-based diagnostic algorithms. Radiography is the next most common modality, and similarly, numerous FDA-approved (Food and Drug Administration) machine learning models exist for the analysis of X-ray images. Optical imaging, primarily microscopy of histological specimens, follows in prevalence. Other modalities such as OCT, ultrasound (US), and thermography are utilized to a much lesser extent. Regarding medical specialties (shown in Figure 2), publications on image analysis methods for neurology, pulmonology, and oncology (excluding brain and lung cancers) are the most prevalent. This is due to the advanced development of these medical fields and the very large number of conditions where the use of diagnostic imaging is essential. The number of described methods for these specialties also correlates with the number of commercially available AI algorithms, which are the most abundant for neurology and pulmonology applications [1]. The number of AI algorithms presented for other specialties is relatively similar and small.

In image analysis tasks, where the primary research problem involves detecting and classifying pathologies or predicting their occurrence, algorithms based on deep learning dominate. It is noteworthy that deep learning methods increasingly incorporate attention mechanisms, which often lead to improved performance. The topic also includes studies employing radiomics, particularly texture analysis [59]. These methods, combined with classical machine learning algorithms or statistical analysis, remain effective tools for biomedical image analysis. However, their popularity is declining in favor of deep learning-based approaches.

For the segmentation tasks described in the topic papers, dedicated deep learning architectures dominate. In particular, the U-Net architecture and its variants are prevalent. Alternative approaches, such as oscillator neural networks, which have proven effective in biomedical image segmentation [60], are virtually absent. The transformer-based models, previously employed in large language models like ChatGPT, are emerging. However, due to the limited number of topic publications utilizing these methods, comparing their performance with classical convolutional neural networks is challenging. The growing popularity of transformer-based algorithms in biomedical image analysis is noteworthy, due to their often-superior performance compared to deep neural networks [24].

## Figures and Tables

**Figure 1:**
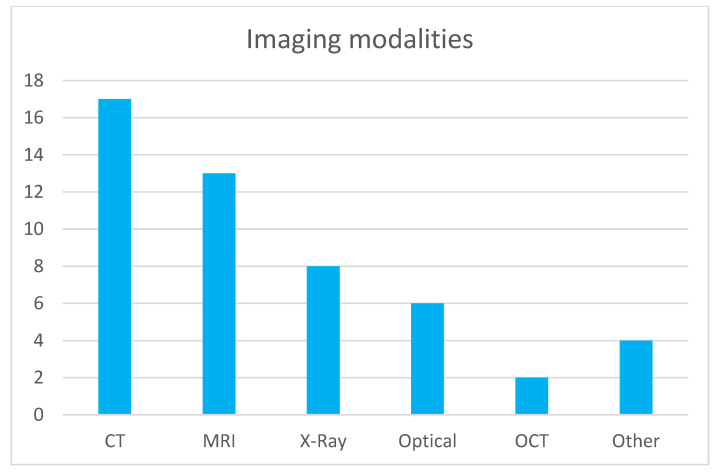
Modality distribution in topic publications.

**Figure 2:**
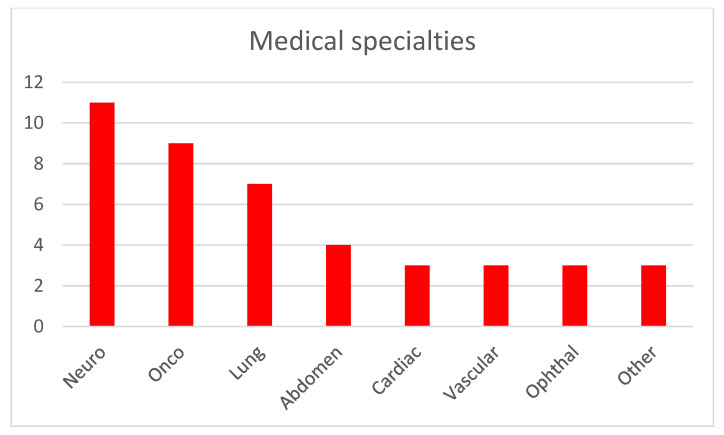
Medical specialties addressed by the described AI methods.

**Table 1:** Summary of medical research using various AI techniques.

AI Technique	Medical Problem
Data classification (CNN)	Chest [2], mammograms [3], tuberculosis [4], brain tumor [5]
Data segmentation (CNN)	Cell counting in H&E-stained images [6], heart and lung anatomy [7], appendix segmentation [8], temporomandibular joints [9], lung fissure [10], COVID-19 diagnosis [11], brain [12], nasolacrimal canal [13], brain tumor [14], osteosarcoma [15], appendix [16], rib fracture [17]
Other applications of CNNs	Detection of anomalies in OCT [18], denoising in chest [19], working with older equipment [20], technical improvements [21], review [1,22,23]
Data analysis with transformers	Spine segmentation [24], bone age estimation [25], analysis of H&E WSI [27], breast [28], brain [29]
Gold standard definitions	Coronary occlusion identification [30], dental caries [31], limb fractures [32], chest [33], OCTA [34], PET [35], scar healing [36], breast cancer [37], discussion helps to apply new technology [38]
ML with radiomics	Lung/prostate tumors [39], PIRADS [41], cell carcinoma [42], prostate cancer [43]
Other applications	Idiopathic macular hole [44], Parkinson’s disease [45], breast cancer [46], dose estimation in CT [47], left atrial volume is equivalent to biplane methods [48], laparoscopy duration [49], osteophytes impact planning of surgical interventions [50], leukemic cells [51], kidney [52]
Unsupervised ML	Active lesions [53], immunohistochemical staining [54]
LLM	Speech recognition [55], describing image content [56,57], detection of acute ischemic stroke [58]

**Table 2:** Summary of topic papers focused on cell or organ segmentation (MAE—mean absolute error, IoU—intersection over union, F1—F1-score, DSC—Dice similarity coefficient, HD—Hausdorff distance, and JAFROC—free-response receiver operating characteristic).

DL Tools	Problem	Modality	Algorithm	Results
CNN [6]	The automatic cell counting on images of H&E-stained slides	H&E-stained images	U-Net	Good agreement between pathologists and AI: MAE(pat) = 13.3%, MAE(AI) = 10.9
CNN [7]	Lung and heart segmentation for cardiothoracic ratio calculation	Chest radiographs	U-Net	Segmentation accuracy: IoU = 0.83, F1 = 0.91
CNN [8]	The accurate segmentation of the appendix	CT	Modified U-Net, DenseNet, Resnet	Segmentation accuracy of modified U-Net: DSC = 0.87, HD = 3.95 mm
CNN [10]	Lung lobe segmentation and lung fissure segmentation	CT	U-Net, Derivative-of-Stick Filter	Segmentation accuracy: F1 = (0.894–0.899), for left and right lung fissures, average DSC = 0.989
CNN [14]	Brain tumor segmentation	MRI	Enhanced by Hierarchical Feature Fusion module	Accuracies of three tumor subregions (enhanced tumor, tumor core, entire tumor): DSC = 88.27%, 91.31%, and 92.96%, respectively
CNN [15]	A novel annotation method for preparing training data for osteosarcoma detection	X-ray	U-Net	Segmentation accuracy for three classes: DSC = 0.644
CNN [16]	Automatic and accurate segmentation of the appendix	CT	Mask R-CNN, Reset101, Grad-CAM	Segmentation accuracy: DSC = 0.87
CNN [17]	Segmentation of rib fractures	Chest radiography	Detectron2 with feature pyramid network	Radiograph with rib fracture classification: AUC = 0.89, rib fracture detection: JAFROC = 0.76
Transformers [24]	The accurate and efficient segmentation of the spine and vertebrae identification	CT	Vertebrae-aware Vision Transformer (a variant of Vision Transformer)	Segmentation accuracy: DSC = (0.95–0.94), IoU = (0.96–0.94) for VerSe2019 and VerSe2020 datasets

**Table 3:** Summary of topic papers focused on pathology detection and classification (AUC—area under the curve).

AI Tools	Problem	Modality	Algorithm	Results
DL [2]	Identifying acute aortic syndrome and thoracic aortic aneurysm in emergency departments	Chest radiographs	InceptionV3, ResNet101, VGG19, nception-ResNet-v2	InceptionV3 F1 = 0.76 for identification of patients with chest pain and suspected acute aortic syndrome/thoracic aortic aneurysm
DL [5]	Brain tumor detection and classification	MRI	Optimized CNN	Accuracy of classification (three tumor types) = 0.97
DL [4]	Identification of pneumonia, COVID-19, and tuberculosis	Chest radiographs	ResNet50, VGG16	ResNet50’s precision and recall rates are close to 0.99 in disease identification
DL with attention module [3]	Computer-aided systems for breast cancer diagnosis in mammograms	X-ray	EfficientNet-b0, cross-mammogram dual-pathway attention module	Classification accuracy = 98.02%, AUC = 0.9664
DL with attention module [5]	Breast cancer diagnosis from histopathology images	Microscope histopathology images	Efficient channel-spatial attention network, improved version of EfficientNetV2	Accuracies of 94.2% at 40×, 92.96% at 100×, 88.41% at 200×, 89.42% at 400× magnifications
DL with attention module [27]	Prediction of lymph node metastasis in colorectal cancer images	H&E-stained whole slide images	Customized deep convolutional neural network attention module, classification module	Accuracy = 0.92, AUC = 0.781–0.824
Radiomics, Statistical analysis [42]	Determination of the grade of cellular differentiation in head and neck squamous cell carcinoma	Multi-Slice Spiral CT	Texture analysis (TA), gray-level co-occurrence matrix	The correlations found between texture parameters and histopathological features suggest that TA is a useful tool in the prognosis and tailoring of treatment strategies for patients with different tumor types
ML/DL, Radiomics [5]	Segmentation and classification of prostate lesions using different ML models	MRI	Texture features, support vector machine, random forest, multiple perceptron, ConvNeXt, ConvNet, ResNet	CNN-based approaches obtained better results compared to the classical machine learning approaches
